# Improvement in distal pancreatectomy for tumors in the body and tail of the pancreas

**DOI:** 10.1186/s12957-021-02159-9

**Published:** 2021-02-15

**Authors:** Li Jiang, Deng Ning, Xiao-ping Chen

**Affiliations:** 1 Department of Biliary and Pancreatic Surgery, Affiliated Tongji Hospital, Tongji Medical College, Huazhong University of Science and Technology, Wuhan, 430030 China; 2 Hepatic Surgery Center, Affiliated Tongji Hospital, Tongji Medical College, Huazhong University of Science and Technology, Wuhan, 430030 China

**Keywords:** Pancreatectomy, Minimally invasive surgical procedure, Pancreatic cancer

## Abstract

**Background:**

Pancreatic resections are complex and technically challenging surgical procedures. They often come with potential limitations to high-volume centers. Distal pancreatectomy is a relatively simple procedure in most cases. It facilitates the development of up-to-date minimally invasive surgical procedures in pancreatic surgery including laparoscopic distal pancreatectomy and robot-assisted distal pancreatectomy.

**Main body:**

To obtain a desirable long-term prognosis, R0 resection and adequate lymphadenectomy are crucial to the surgical management of pancreatic cancer, and they demand standard procedure and multi-visceral resection if necessary. With respect to combined organ resection, progress has been made in evaluating and determining when and how to preserve the spleen. The postoperative pancreatic fistula, however, remains the most significant complication of distal pancreatectomy, with a rather high incidence. In addition, a safe closure of the pancreatic remnant persists as an area of concern. Therefore, much efforts that focus on the management of the pancreatic stump have been made to mitigate morbidity.

**Conclusion:**

This review summarized the historical development of the techniques for pancreatic resections in recent years and describes the progress. The review eventually looked into the controversies regarding distal pancreatectomy for tumors in the body and tail of the pancreas.

## Introduction

Distal pancreatectomy (DP) emerged as the standard surgical procedure for the treatment of pancreatic body and tail lesions since the first case of pancreatic body and tail resection in 1884 [1]. With the advancement in technology, the revolution leaped from laparotomy to laparoscopic and now robotic-assisted pancreatectomy. Regarding malignant tumors in the body or tail of the pancreas, methods that are consistent with the principles of oncology for achieving a precise prognosis have been explored for decades [2, 3]. As the concept of precise surgical treatment is upgrading, spleen preservation during DP is becoming increasingly appreciated. Despite the progress in the promising therapeutic strategies, postoperative pancreatic fistula (POPF) remains a surgical complication that begs for more interventions. Thus, this study aimed to review the surgical progress and controversial issues related to DP.

## Development of minimally invasive technology in distal pancreatectomy

### Evolution from open distal pancreatectomy to laparoscopic distal pancreatectomy

Implementation of minimally invasive techniques in pancreatic surgery was once restrained by anatomic disadvantages. These include complex proximity of the pancreas to major vasculature and its retroperitoneal location [4].

The first laparoscopic DP (LDP) was reported by Cuschieri et al. in 1996 following the successful treatment of chronic pancreatitis. This confirmed that a minimally invasive technique was feasible for left pancreatic surgery [5]. A decade later, the rapid development of minimally invasive techniques put LDP at the forefront and has been increasingly implemented as an alternative to open surgery [6]. Despite the advantages over the open approach, the minimally invasive approach suffers some adoption issues for pancreatic surgery. With the evolution in technology and experience, the standard approach to lesions of the pancreatic body and tail based on the existing literature is laparoscopic distal or left-sided pancreatic resections [7].

With its widespread use worldwide, LDP was proven to be associated with improved perioperative outcomes, including less intraoperative blood loss and shorter length of hospital stay, compared to open distal pancreatectomy (ODP). Patients who had laparoscopic distal pancreatectomy experience shorter hospital stay compared to patients undergoing open resection. In addition, even though with laparoscopic distal pancreatectomy, patients experience lesser blood loss, the selection is imperative because patients who required conversion might face higher rates of complications and pancreatic leak [8–14]. Reduced postoperative morbidity was also shown with LDP from the emerging literature [8, 13, 14]. However, others suggested that there was no significant difference in overall complications, including POPF, between LDP and ODP [9–12].

Thus far, two randomized controlled trials (RCTs) comparing LDP and ODP have been reported in the current literature. A single-blind multicenter RCT (Laparoscopic versus Open Pancreatoduodenectomy for Pancreatic or Periampullary Tumors (LEOPARD)) study was reported by de Rooij. Patients with pancreatic body or tail tumors without vascular invasion were randomly divided into the minimally invasive distal pancreatectomy (MIDP) and ODP groups. The results revealed reduced blood loss and longer operating time during MIDP [15]. Another study was unblinded, parallel-group, single-center superiority trial reported by Björnsson. This study established similar advantages in terms of intraoperative blood loss and postoperative hospital stay in the LDP group [16]. Based on the findings of the two RCTs, MIDP is found to shorten the time to functional recovery and improve the quality of life without increasing the cost of hospitalization as compared with ODP. Surprisingly, both RCTs failed to demonstrate the advantage of LDP on morbidity. To address the reduction in major morbidity observed during the LEOPARD trial, which was deemed statistically insignificant, Klompmaker et al. conducted an international multicenter study to increase the sample patient population. They performed appropriate adjustments for the confounding factors by indication. Their results suggested that MIDP was independently associated with reduced composite major morbidity, which includes death and severe complications [17]. With robotic-assisted procedures, a significant increase in the frequency of resections was performed for asymptomatic tumors. This is putting technology far front and a promising intervention in the nearest future.

### Mini-invasive procedure in pancreatic ductal adenocarcinoma

As LDP improves short-term prognosis over ODP, it has been proposed that LDP can yield the same or non-inferior oncologic outcome compared with ODP. This includes similar surgical metrics such as the rate of R0 resection and long-term survival or disease-free survival [7, 11]. Subsequently, the safety and efficacy of LDP were corroborated by a few studies on LDP for pancreatic neuroendocrine tumors (PNETs). In one of these studies, patients who underwent MIDP and ODP for the treatment of PNETs had comparable oncologic outcomes, including long-term disease-free survival and overall survival [13, 14, 18, 19]. Although the ODP group might have had higher lymph node yield when compared with the MIDP group, long-term survival was not influenced by the choice of the surgical option [10, 14]. Therefore, LDP is advantageous for benign and low-grade malignant pancreatic lesions as compared to traditional open surgery, with the same oncological efficacy (Table 1).

**Table 1:** Short-term postoperative outcomes of laparoscopic distal pancreatectomy and open distal pancreatectomy

Author, year	Research type	Research classification (laparoscopic/robotic vs. laparotomy)	Postoperative pancreatic fistula (%)	Complications (%)	Hospitalization time (days)	Mortality (%)
Kooby, 2008 [7]	Retrospective	142 vs. 200	11 vs. 18	40 vs. 57	5.9 ± 3.8 vs. 9.0 ± 6.0^A^	0 vs. 1
Vijan, 2010 [9]	Retrospective	100 vs. 100†	17 vs. 17	34 vs. 29	6.1 ± 2.4 vs. 8.6 ± 5.9^A^	3 vs. 1
Limongelli, 2012 [12]	Retrospective	16 vs. 29	18 vs. 20	4 vs. 12	6.4 ± 2.3 vs. 8.6 ± 1.7^A^	0 vs. 1
Stauffer, 2013 [11]	Retrospective	82 vs. 90	6 vs. 10	13 vs. 20	4 (1–10) vs. 8 (3–18)^B^	N/A
Lee, 2015 [10]	Retrospective	131/37 vs. 637	6/5 vs. 9	32/32 vs. 40	5 (5–7)/5 (5–8) vs. 7 (6–9)^B^	0.6/0 vs. 0
Xourafas, 2015 [13]	Retrospective	73 vs. 98	6 vs. 15	30 vs. 47	5 (3–18) vs. 7 (4–39)^C^	5 vs. 13
Han, 2018 [18]	Retrospective	42 vs. 52	9.5 vs. 15.4	26.2 vs. 34.6	7 (4–18) vs. 9 (7–66)^D^	N/A
Zhang, 2019 [19]	Propensity score-matched	141 vs. 141	10.6 vs. 9.9	48.2 vs. 58.2	4 (4–6) vs. 7 (5–9)^C^	0.7 vs. 1.4
De Rooij, 2019 [15]	Randomized control trial	51 vs. 57	39 vs. 23	25 vs. 38	6 (4–13) vs. 8 (6–12)^B^	0 vs. 2
Klompmaker, 2019 [17]	Retrospective	1562 vs. 1359	N/A	22.4 vs. 33	N/A	0.6 vs. 1
Partelli, 2020 [14]	Retrospective	40 vs. 84	50 vs. 55	62.5 vs. 75	7.5 (6–9) vs. 9 (8–11)^B^	0 vs. 0
Bjornsson, 2020 [16]	Randomized control trial	29 vs. 29	31 vs. 38	N/A	5 (4–5) vs. 6 (5–7)^B^	N/A

*N/A* not available

^A^*P* < 0.001; results expressed as mean ± standard deviation

^B^*P* < 0.001; results expressed as median (interquartile range)

^C^*P* < 0.05; results expressed as median (range)

^D^*P* < 0.001; postoperative hospital days, expressed as median (range)

^†^Data after cohorts were matched

In the recent past, it was believed that laparoscopic radical surgery for pancreatic cancer was too difficult for popularization due to its associated technical challenges [20]. Furthermore, due to these challenges, the oncologic outcome might be questionable [21]. However, with the recent advancement in technology and extensive development of comprehensive treatment options, considerable progress has been made with LDP in the treatment of pancreatic cancer.

In 2010, Kooby reported the first series of 235 patients with pancreatic ductal adenocarcinomas in a multicenter analysis that compared ODP with LDP. The results showed no significant difference in the positive margin rate, number of positive lymph nodes, and overall survival rate between the two groups. Logistic regression analysis suggested that the prognosis had no correlation with the surgical technique (ODP vs. LDP). This indicates that minimally invasive techniques may not affect the prognosis of tumors [22]. Following studies on early-stage pancreatic cancer cases, similar results were reported. This verified that LDP was comparable to ODP in terms of safety and effectiveness in the treatment of pancreatic cancer [23, 24]. Other large series further proved that patients with pancreatic cancer who underwent LDP had faster recovery of gastrointestinal function, shorter hospital stay, less intraoperative blood loss, and fewer overall complications than those who had open procedure. Meanwhile, the incidence of grade C pancreatic fistula was lower in the LDP group, and no significant difference in oncologic surgical indices and tumor prognosis was found between the two groups [25–29].

Recently, a pan-European propensity score-matched study (Minimally Invasive versus Open Distal Pancreatectomy for Ductal Adenocarcinoma (DIPLOMA) which includes patients with pancreatic ductal adenocarcinomas from 34 centers in 11 countries was reported by van Hilst et al. They found thatblood loss was less and hospital stay was shorter after MIDP, while morbidity and mortality had no correlation with the choice of the surgical technique. Higher R0 resection rate and lower lymph node retrieval in MIDP did not influence the comparable survival for pancreatic ductal adenocarcinoma after MIDP and ODP [30].

Overall, LDP is believed to be safer and more effective than traditional open surgery in some selected cases of pancreatic cancer, in terms of oncologic outcome (Table 2). With few cases and limited evidence in the literature, further prospective RCT interventions are imperative.

**Table 2:** Comparison of oncological outcomes between laparoscopic distal pancreatectomy and open distal pancreatectomy for pancreatic cancer

Author, year	Research type	Study classification (laparoscopic/robotic vs. laparotomy)	Number of lymph nodes detected (*n*)	Negative margin rate (%)	Median survival time (months)
Kooby, 2010 [22]	Retrospective	23 vs. 189	13.8 ± 8.4 vs. 12.5 ± 8.5 A	74 vs. 73	16 vs. 16
Magge, 2013 [23]	Retrospective	28 vs. 34	11 (8–20) vs. 12 (6–19) B	86 vs. 88	19
Rehman, 2014 [28]	Retrospective	8 vs. 14	16 (1–27) vs. 14 (0–26) C	88 vs. 86	33 vs. 52
Shin, 2015 [25]	Retrospective	70 vs. 80	12 (1–34) vs. 10 (1–64) C	75.7 vs. 83.8	33.4 vs. 29.1
Lee, 2015 [10]	Retrospective	23 vs. 249	11 ± 7.6 vs. 15 ± 8.7 A	100 vs. 88	N/A
Sharpe, 2015 [29]	Retrospective	144 vs. 625	14.9 ± 10.0 vs. 13.3 ± 9.9 A	87 vs. 78	N/A
Stauffer, 2016 [26]	Retrospective	44 vs. 28	25.9 (5–48) vs. 12.7 (1–45) B**	95.5 vs. 82.1	26.6 vs. 26.4
Bauman, 2017 [24]	Retrospective	33 vs. 46	14.5 ± 1.1 vs. 17.5 ± 1.2 A	77 vs. 87	18 vs. 15
Raoof, 2018 [27]	Propensity score-matched	563 vs. 563	12 (6–18·5) vs. 22 (14–31) B**	85.1 vs. 81.5	N/A
Van Hilst, 2019 [30]	Propensity score-matched	340 vs. 340	14 (8–22) vs. 22 (14–31) B*	67 vs. 58	28 vs. 31

*N/A* not available

A. Results expressed as mean ± standard deviation

B. Results expressed as median (interquartile range)

C. Results expressed as median (range)

*P = 0.05; **P = 0.0001

### Development of robot-assisted distal pancreatectomy

In 2002, Melvin et al. reported the first case of robot-assisted pancreatic body and tail resection [31]. In the following two decades, robotic pancreatic surgery became increasingly common worldwide, especially in North America [32]. It has been well known that robotic distal pancreatectomy (RDP) is related to higher spleen preservation rate and less blood loss than LDP, while morbidity and mortality are similar between RDP and LDP [33–36].

As RDP is increasingly being performed across centers in the USA [37], Raoof et al. reported a national comparison of RDP and LDP with respect to the oncologic outcomes of pancreatic cancer in 2018. They found no difference in the margin-positive rate, lymph node yield, hospital stay, and overall survival between the two groups [38]. Moreover, the most recent report on pancreatic ductal adenocarcinomas in a national cohort by Nassour et al. suggested improved long-term overall survival for RDP than for ODP [39].

The significant cost of robotic instrumentation renders RDP more expensive than LDP; hence, this approach is not cost-effective [40]. De Pastena et al. reported that RDP could become more cost-effective than laparoscopic procedure if there is a willingness to pay for additional 4800 euros per quality-adjusted life-year [41]. Overall, caution should be taken in justifying the use of robotic techniques in the current health care environment.

## Advances in surgical procedures: radical resection of pancreatic body or tail cancer

Pancreatic cancer is difficult to diagnose in the early stages because of its rapid progression. About 20–30% of the cancer cases occur in the body and tail of the pancreas [2]. As radical R0 resection and regional lymph node dissection ensure better long-term prognosis, methods to achieve this goal through standardized surgical methods have been a popular topic in recent years.

### Radical antegrade modular pancreatosplenectomy

Steven M. Strasberg proposed radical antegrade modular pancreatosplenectomy (RAMPS) in 2003 to address the limitations of traditional surgery for pancreatic body and tail cancer. Positive margins caused by insufficient retroperitoneal dissection, poor control of intraoperative bleeding, and inadequate lymphadenectomy were established [42]. Contrary to the traditional radical surgery for tumors located in the body or tail of the pancreas, RAMPS adopts the “medial to lateral” approach. First, the neck of the pancreas is transected, followed by ligation of the splenic artery and vein at the root, with dissection of the left lymph nodes of the celiac trunk and superior mesenteric artery. Then, the whole sample (including the body and tail of the pancreas, spleen, and left anterior renal fascia) is resected along the surface of the left renal vein.

Subsequently, the safety and efficacy of RAMPS were further demonstrated by Mitchem et al. in 2012. They retrospectively analyzed the data of patients from 1999 to 2008 and found out that the R0 resection rate and 5-year survival rate were satisfying. Compared with the traditional surgery, the results of RAMPS have been greatly improved [43]. Other large series have also suggested that RAMPS can increase the total number of retrieved lymph nodes and the rate of negative margin; thus, it will facilitate radical resection and prolong survival [44].

As a modification of standard retrograde pancreatosplenectomy, RAMPS was compared with standard retrograde pancreatosplenectomy in a report by Abe et al. The short-term outcomes revealed less intraoperative blood loss and shorter operating time with RAMPS, as well as better lymph node retrieval. Furthermore, more frequent R0 resection as compared with standard retrograde pancreatosplenectomy was also revealed. Moreover, RAMPS offered improved survival over standard retrograde pancreatosplenectomy [45].

Exposure of the dissection plane around the deep organs under the left costal margin is often technically challenging. This can increase the risk of inadequate oncologic dissection. To address this, Watanabe et al. suggested the left kidney mobilization approach. This proved to be safe and oncologically sound in lateral retroperitoneal dissection during RAMPS for distal pancreatic cancer [46].

As RAMPS has yielded improved oncologic outcomes for all tumors of the pancreas, except for pancreatic cancer, Sivasanker et al. suggested that RAMPS should be considered for all operable tumors involving the body and tail of the pancreas, when a preoperative histologic diagnosis is not available [47].

### Appleby and distal pancreatectomy with en bloc celiac axis resection

For locally advanced pancreatic cancer located in the body and tail with a partial invasion of the peripheral blood vessels but no distant metastasis, surgical resection can prolong survival. With the idea of “borderline resectable” being widely accepted and the advancement of surgical technology [2, 3], many reports have suggested that en bloc vascular resection is safe and feasible. It also enables R0 resection in some locally advanced pancreatic cancer cases and beneficial to the patients [48–50].

Appleby initially described radical gastrectomy combined with celiac trunk resection in 1952 for locally advanced gastric cancer [51]. The Appleby procedure consisted of multi-organ resections, including the celiac trunk, stomach, distal pancreas, and spleen. Following decades of progress, Hirano and Kondo reported a modified Appleby procedure in 2007, namely distal pancreatectomy with celiac axis resection (DP-CAR). This is a surgical procedure for pancreatic body and tail cancer that invades adjacent organs and requires combined celiac trunk resection. It is a treatment option for selected patients with pancreatic cancer involving the celiac axis. When options are given for acceptable 90-day mortality and overall survival, DP-CAR is the choice. With the combination of vessel reconstruction to achieve curative resection, the quality of life of patients after surgery is thus improved by preserving the entire stomach [52]. This approach is complicated and must be performed by experienced surgeons. Preserving the left gastric artery during DP-CAR, as reported by Kimura and Okada et al., may reduce the incidence of complications such as delayed gastric emptying [53, 54]. From one of the recent studies [54], DP-CAR may reduce the risk of ischemic complications with preservation or reconstruction of the LGA. Apparently, this study was found in consistence with safe and rational procedure for locally advanced pancreatic cancer of DP-CAR. Other large series have also shown that this modified Appleby procedure is safe and feasible and can relieve intractable pain in pancreatic cancer patients. This is possible partly due to the removal of the peripancreatic and retroperitoneal nerve plexus [55].

With the extensive development in recent years, neoadjuvant therapy has been proven to contribute to overall survival in pancreatic cancer [56]. The neoadjuvant FOLFIRINOX (folinic acid, fluorouracil, irinotecan, and oxaliplatin) regimen may eventually downstage the tumor. This renders locally advanced pancreatic ductal adenocarcinoma resectable and increasing the application of DP-CAR for better survival. Furthermore, due to the safe and rational procedure that DP-CAR comes with, the procedure can be performed without preoperative embolization of the common hepatic artery CHA. In the meantime, neoadjuvant chemotherapy can enable the identification of occult micrometastasis before patients are committed to DP-CAR [57].

Recent reports corroborated that DP-CAR following neoadjuvant chemotherapy was associated with a better prognosis than and similar morbidity and mortality to classic DP. The combination of neoadjuvant chemotherapy and DP-CAR may therefore be a beneficial and safe therapeutic strategy for patients with locally advanced pancreatic cancer [58, 59]. However, this should be performed with great caution because of high morbidity. For DP with splenectomy (DP-S) patients (high risk of positive surgical margins), DP-CAR is an option [60].

Recently, it is hypothesized that, depending on the tumor progression, it might influence the prognosis in patients who undergo DP-CAR. To address this from happening, a combination of left inferior phrenic artery (IPA) and left gastric artery (LGA) resection is required. This was a significant risk factor for ischemic gastropathy IG. Among other independent factors that might serve as confounding during the study involving DP-CAR are but not limited to longer operative time, adjuvant chemotherapy, and higher age [61]. These require careful study to ascertain if they are good independent predictors of survival.

## Renewal of operative concept: spleen preservation

### Choice of spleen preservation

Traditionally, DP is often combined with splenectomy due to the proximal anatomical relationship of the pancreas and the splenic vasculature. In recent years, with a better understanding of splenic function, it has been well accepted that splenectomy may increase the risk of postoperative complications. These include hypercoagulability, overwhelming post-splenectomy infection, and poor cancer survival [62]. Therefore, it was proposed that the spleen should be preserved during DP for benign, borderline, and low-grade malignant tumors, to lower the rate of postoperative complications and to improve the quality of life of patients after DP [63, 64]. Recently, two meta-analyses verified that spleen-preserving distal pancreatectomy for benign and low-grade malignant tumors is a safe procedure. This is associated with better short-term outcomes than distal pancreatosplenectomy [65, 66]. The standard procedure for body and tail pancreatic tumors is laparoscopic distal pancreatectomy with splenectomy. This procedure comes with technical difficulties and due to that concomitant splenectomy is often performed. A reduced surgical site infection (SSI) and overall complications may result with spleen-preserving laparoscopic distal pancreatectomy while further studies are needed for long-term outcomes [67]. The effect of spleen-preserving total gastrectomy on postoperative infectious complications study reported a strong association with decreased postoperative infectious complications. While the overall morbidity is also low, a potential evidence-based treatment for gastric cancer is revealed [68].

As splenectomy was traditionally performed during DP for malignant diseases, it was thought to be mandatory for radical lymphadenectomy and negative margin [69]. Spleen preservation in patients with pancreatic cancer can be potentially beneficial in terms of cancer recurrence after oncological surgery because of the important role plays by the spleen in the immune system. Some researchers found that the incidence of splenic hilar lymph node metastasis was non-existent or low enough for spleen-preserving radical DP to be taken into consideration [70]. Recently, Collard et al. reported that lymph nodes located in station 10 (splenic hilum) were always metastasis-free in left-sided pancreatic ductal adenocarcinoma cases. The tumoral involvement of the spleen or splenic hilum was observed in only 8% of cases, which could be accurately predicted by computed tomography and intraoperative findings [71]. The results of this study therefore suggest that routine splenectomy may not be mandatory during DP for pancreatic ductal adenocarcinoma.

### Preservation or resection of the vessels

Spleen-preserving distal pancreatectomy with ligation of the splenic artery and vein was first described in 1988 by Warshaw. It has been well accepted as the procedure of choice for spleen-preserving distal pancreatectomy. Warshaw’s procedure involves resection of the splenic artery and vein along with the pancreas while sparing the short gastric vessels and left gastroepiploic vessels as a collateral supply for the spleen. Splenic vascular disconnection was reported to lead to a series of complications (such as splenic infarction, gastric varices, and even left-sided regional portal hypertension) [72]. Subsequent series found that the rate of the complications could be minimized to an acceptable level that will minimize adverse clinical consequences [73, 74]. Furthermore, in 2011, the Warshaw group reported a long-term follow-up result for the Warshaw operation. Among 125 surgical patients who were followed up for up to 21 years, only 1.9% of them required a reoperation because of splenic infarction. While perigastric varices developed in 16 of 65 (25%) patients who had evaluable postoperative imaging during follow-up, none of the patients develop gastrointestinal bleeding or segmental hypersplenism [75].

Spleen-preserving distal pancreatectomy can also be performed by sparing the splenic vessels. As splenic vessel-preserving approach has long been used in the management of pancreatic trauma [76]. Kimura et al. modified the technique and reported first in 1996 its application to benign lesions of the left pancreas [77]. Splenic vessel-preserving DP is time-consuming and technically challenging due to the fact that the splenic vein is often embedded in the sulcus of the posterior side of the pancreatic body. Therefore, the operative duration of spleen-preserving distal pancreatectomy is increased and associated with increased intraoperative blood loss as compared to Warshaw’s procedure [78–80].

Although it was well accepted that splenic vessel-preserving DP can reduce spleen-related complications due to conservation of the splenic vessel, compared to Warshaw’s technique, splenic vessels may fail to remain patent as a result of thermal damage by ultrasonic shear or vascular manipulations during the surgery. Therefore, some researchers have suggested that it would be beneficial to convert to Warshaw’s procedure during the splenic vessel-preserving DP surgery if the dissection for conserving the splenic vessels is anticipated to be difficult and require frequent vascular manipulation [79, 81].

With the development of minimally invasive technique, the laparoscopic approach made splenic vessel preservation and Warshaw’s procedure safer and more feasible than previous [80, 82]. Adam et al. reported in 2013 the largest series of DP with splenic conservation by laparoscopic technique, including 140 patients with or without splenic vessel preservation. After comparison of Warshaw’s technique with laparoscopic splenic vessel preservation with respect to the intention-to-treat principle, both techniques were found to be efficient and similar in terms of the rates of overall morbidity and POPF, blood loss, and operative time. They suggested that laparoscopic splenic vessel preservation should be attempted whenever possible and switched to Warshaw’s procedure in cases in which ① the tumor is located close to the splenic hilum, ② the vessels are embedded in the pancreatic parenchyma, and ③ local inflammation is present [74].

In recent years, spleen preservation in DP was done via robotic technique. The result indicated that both vessel-conserving technique and Warshaw’s technique can be used safely to achieve an increased spleen-preserving rate during robot-assisted surgery [83, 84].

Overall, the multiple methods and techniques provide more choices for spleen-preserving procedure, which further improves the prognosis of the surgery.

## Management of pancreatic stump

Among complications after DP, clinically relevant POPF is generally considered to be the leading cause of morbidity, with a frequency of 3–45% [85]. Various mitigation strategies were advocated for by different groups. Among these, most focused on optimizing management of the pancreatic remnant. Established management procedures for pancreatic remnant after DP include hand-sewn suture or stapler closure and pancreatico-enteric anastomosis. However, some controversies remain regarding the selection of the ideal approach.

### Closure of the pancreatic stump

Hand-sewn closure of the remnant stump is the classical procedure during left pancreatectomy, and stapled transection of the pancreas has become increasingly popular. The Distal Pancreatectomy (DISPACT) multicenter RCT in 21 European centers showed no significant difference in the incidence of pancreatic fistula between the hand-sewn group and the stapler group [86]. These findings were further corroborated by a meta-analysis in 2015 [87]. Most recently, however, a multinational retrospective study of 2026 left pancreatectomies from 10 institutions reported statistically significant with a decreased POPF rate by using the stapler technique compared with hand-sewn closure [88]. As the debate progresses, it may be appropriate for surgeons to persist with their preferred surgical approach.

The reinforced stapler technique has been studied extensively in recent years. An RCT conducted in 2012 showed that reinforcement of stapled pancreatic transection using mesh buttress decreases the incidence of POPF for DP [89]. However, a meta-analysis and a recent RCT suggested otherwise [90, 91]. The recent multicenter RCT from nine hospitals conducted in Japan revealed a reinforced stapler with a polyglycolic acid membrane for pancreatic transection during DP. This does not reduce the incidence of CR-POP clinically relevant POPF compared to stapler without reinforcement. While the role of reinforced stapler remains unclear, the peri-firing compression technique has been widely accepted as a procedure that is associated with lower clinically relevant POPF rate after stapling [92].

Coverage of the pancreatic stump by additional biologic sealant or patches has been proposed to be protective in terms of POPF. Nevertheless, recent reports suggested that neither biologic sealant nor biologic patch provides relevant benefits with respect to POPF after DP [93]. Meanwhile, the DIStal resection of the pancreas with or without COVERage of the pancreatic remnant (DISCOVER) randomized trial showed that additional coverage of the pancreatic stump with an autologous patch could decrease the POPF rate after left pancreatectomy [94]. This conclusion was in consistence with a more recent network of meta-analysis [95]. From study on subgroup analysis, management of drains after distal pancreaticoduodenectomy found inconclusive remarks. Only something is safer than nothing to routine peritoneal drainage. Furthermore, the patients who underwent DP can attempt to omit the drainage [96].

### Pancreatic-digestive tract anastomosis

The “Achilles heel” of the modern day, the pancreatico-enteric anastomos is single-stage, pancreatoduodenectomy. The function of the Oddi sphincter is preserved during DP, which leads to high intrapancreatic duct pressure that predisposes to failure of closure of the pancreatic stump. Pancreaticojejunostomy for the pancreatic stump can restore the drainage of pancreatic fluid to the intestine and alleviate the intrapancreatic duct pressure. A meta-analysis showed that pancreaticojejunostomy for the pancreatic remnant after DP significantly reduces POPF rates more than suture closure [97]. On the other hand, the only RCT of pancreaticojejunostomy vs. stapler closure failed to demonstrate the benefits of pancreaticojejunostomy of pancreatic remnant over stapler during DP in terms of POPF. This may be due to the fact that the optimal procedure for pancreaticojejunostomy remains controversial. To address this issue, we introduced a new pancreaticojejunostomy technique for DP—invaginated end-to-end pancreaticojejunostomy with transpancreatic transverse U-sutures—that was first applied in pancreaticoduodenectomy [98]. In a series of DP in our institute, we used this technique to manage the pancreatic remnant in 18 patients, with only two patients developing grade A-type POPF and none developing grade B/C-type POPF [99]. The result of this pilot study therefore seems promising. In achieving the best outcomes, adherence to the tenets of anastomotic reconstruction is the mainstay. In addition, the performance of a safe and reproducible anastomosis with a low clinically relevant POPF rate should be considered. During the need to perform a high-risk pancreatico-enteric anastomosis, appropriate selection and use of fistula mitigation strategies may help provide optimal outcomes. Binding pancreaticojejunostomy and the use of external stents will give intresting results. Besides the choice of techniques, other parameters like surgical volume, meticulous surgical technique, and other management parameters determine the successful management of pancreatic anastomoses [100].

## Conclusion

With the revolutionalization of minimally invasive surgical technique, LDP has been proven to yield a better short-term prognosis over ODP, and the development of RDP may provide a further promising long-term prognosis for pancreatic cancer cases. To acquire R0 resection and adequate lymphadenectomy for desirable long-term prognosis, routine procedures have been explored to fulfill the en bloc resection in pancreatic cancer surgery, such as RAMPS, Appleby, and DP-CAR. Since multi-visceral resection is believed to be necessary in certain cancer patients, spleen-preserving procedures improve the prognosis in benign and borderline tumor cases, even in selected cancer cases. Despite the constantly improved surgical approaches for DP, current techniques for pancreatic stump management needs to be further evaluated due to the high incidence of POPF. Since retrieved literatures from the database are mostly retrospective in nature, supplementing with prospective and randomized controlled studies will ascertain and validate these results.

## Data Availability

The datasets used and/or analyzed during the current study are available from the corresponding author on reasonable request.
